# Microstructural Damage of the Posterior Corpus Callosum Contributes to the Clinical Severity of Neglect

**DOI:** 10.1371/journal.pone.0048079

**Published:** 2012-10-24

**Authors:** Marco Bozzali, Chiara Mastropasqua, Mara Cercignani, Giovanni Giulietti, Sonia Bonnì, Carlo Caltagirone, Giacomo Koch

**Affiliations:** 1 Neuroimaging Laboratory, Santa Lucia Foundation Istituto Di Ricovero e Cura a Carattere Scientifico, Rome, Italy; 2 Brighton & Sussex Medical School, Clinical Imaging Sciences Centre, Falmer, United Kingdom; 3 Department of Clinical and Behavioural Neurology, Santa Lucia Foundation, Istituto Di Ricovero e Cura a Carattere Scientifico, Rome, Italy; 4 Department of Neuroscience, University of Rome ‘Tor Vergata’, Rome, Italy; 5 Stroke Unit, Department of Neuroscience, University of Rome Tor Vergata, Rome, Italy; West China Hospital of Sichuan University, China

## Abstract

One theory to account for neglect symptoms in patients with right focal damage invokes a release of inhibition of the right parietal cortex over the left parieto-frontal circuits, by disconnection mechanism. This theory is supported by transcranial magnetic stimulation studies showing the existence of asymmetric inhibitory interactions between the left and right posterior parietal cortex, with a right hemispheric advantage. These inhibitory mechanisms are mediated by direct transcallosal projections located in the posterior portions of the corpus callosum. The current study, using diffusion imaging and tract-based spatial statistics (TBSS), aims at assessing, in a data-driven fashion, the contribution of structural disconnection between hemispheres in determining the presence and severity of neglect. Eleven patients with right acute stroke and 11 healthy matched controls underwent MRI at 3T, including diffusion imaging, and T1-weighted volumes. TBSS was modified to account for the presence of the lesion and used to assess the presence and extension of changes in diffusion indices of microscopic white matter integrity in the left hemisphere of patients compared to controls, and to investigate, by correlation analysis, whether this damage might account for the presence and severity of patients' neglect, as assessed by the Behavioural Inattention Test (BIT). None of the patients had any macroscopic abnormality in the left hemisphere; however, 3 cases were discarded due to image artefacts in the MRI data. Conversely, TBSS analysis revealed widespread changes in diffusion indices in most of their left hemisphere tracts, with a predominant involvement of the corpus callosum and its projections on the parietal white matter. A region of association between patients' scores at BIT and brain FA values was found in the posterior part of the corpus callosum. This study strongly supports the hypothesis of a major role of structural disconnection between the right and left parietal cortex in determining ‘neglect’.

## Introduction

Neglect is clinically defined as the impaired or lost ability to react to or process sensory stimuli when presented in the hemispace contralateral to a brain lesion due to acute stroke, in the absence of any remarkable sensory loss [1], [2], [3], [4]. This condition is frequently observed in the case of an acute/sub-acute damage affecting the right hemisphere (RH), while, in contrast, neglect symptoms are rarely observed after damage localized to the left hemisphere (LH) [5]. According to previous literature, neglect has an incidence of about 45% in acute strokes of the RH, and persistent deficits are observed in one third of cases [6]. Despite its clinical relevance, the pathophysiology of neglect is still poorly understood. Two major hypotheses have been formulated so far. One is based on the assumption that the RH controls attention orienting in both left and right hemispace, while the LH controls the direction of attention in the right hemispace only (i.e., “hemispheric specialization” hypothesis) [7], [8]. This hypothesis is supported by the far greater prevalence of neglect following RH than LH damage, as well as by imaging studies demonstrating a greater extent of activations in the RH than in the LH during tasks involving shifts of visuo-spatial attention [9], [10], [11]. Alternatively, Kinsbourne's theory has proposed a mechanism of hemispheric rivalry [12]. This second hypothesis assumes that an asymmetric dynamic balance exists between parieto-frontal circuits in the two hemispheres, with the RH prevailing over the LH (i.e., hemispheric competition hypothesis) [12]. Each hemisphere is thought to be responsible for orienting attention toward the contralateral hemi-space and to control the contralateral hemisphere trough mechanisms of reciprocal inhibition, with a right hemispheric prevalence in inhibiting the LH (for a recent review see Koch et al., [13]). This theory is supported by clinical evidence that patients with extinction often manifest directional biases, favoring stimuli that are relatively ipsi-lesional over those which are relatively contra-lesional within and between visual fields. In other words, the excessive attention to the right hemispace (at the expense of the left hemispace) as due to reduced inhibition of the left hemisphere would lead to clinically detectable neglect. In support to the hemispheric competition hypothesis, we have recently demonstrated that the right, but not the left human posterior parietal cortex (PPC) exerts a strong inhibitory activity over the contralateral homologous area by a short-latency connection, using a combined method of trifocal transcranial magnetic stimulation (TMS) and diffusion MRI [14]. Notably, we demonstrated that this interaction is mediated by direct transcallosal projections located in the posterior portion of the corpus callosum through callosal fibers crossing the regions IV and V [14]. These data suggest that this anatomo-functional network might represent a possible neurophysiological basis for interhemispheric functional asymmetry. However, to be confirmed, this interpretation requires a direct demonstration that, in patients with a right parietal lesion and neglect, the posterior part of the corpus callosum and its projections to the LH are microscopically damaged (anatomical disconnection) in the absence of macroscopic abnormalities. Further, this anatomical disconnection should be associated with the presence and severity of neglect. Diffusion imaging provides a unique form of magnetic resonance imaging (MRI) contrast that enables the diffusional motion of water molecules to be measured and, as a consequence of the interactions between tissue water and cellular structures, provides information about the size, shape, orientation and geometry of brain structures [15]. Pathological abnormalities that modify tissue integrity, including microscopic degeneration of white matter fibres, can result in an altered diffusion coefficient, which can be measured *in vivo* using MRI. Diffusion imaging has already been demonstrated, in several neurological and psychiatric conditions (e.g., [16], [17], [18], [19]), to be capable of detecting and quantifying subtle white matter changes. For instance, microscopic abnormalities have been reported in the normal appearing white matter of patients with neurodegenerative dementia, and strict associations with clinical data have been found with patients' cognitive disabilities [20], [21]. There are many possible methods to analyze diffusion imaging data. One can choose a set of anatomical locations *a priori*, and derive from them quantitative measures of microscopic integrity, such as fractional anisotropy (FA) and mean diffusivity (MD) [16]. Alternative and more intriguing methods of image analysis are the so called voxel-wise approaches, which do not require any *a priori* hypothesis on the anatomical localization of white matter abnormalities. Tract-Based Spatial Statistics (TBSS) [22], introduced by Smith and collaborators, is one of the most popular of these methods. It allows testing for group comparisons of regional FA (i.e., a quantity that has traditionally been interpreted as an index of microscopic tissue integrity) as well as for correlations between this quantity and clinical or neuropsychological variables. The same procedure can be applied to other diffusion indices, such as MD (an orientation averaged measure of magnitude of diffusion), axial diffusivity (Dax, the diffusion coefficient along the direction of maximum diffusion) and radial diffusivity (Drad, the diffusion coefficient in the direction perpendicular to the maximum). These additional quantities can help in the interpretation of the results in terms of pathological substrate of the FA changes [23 24].

**Figure 1:**
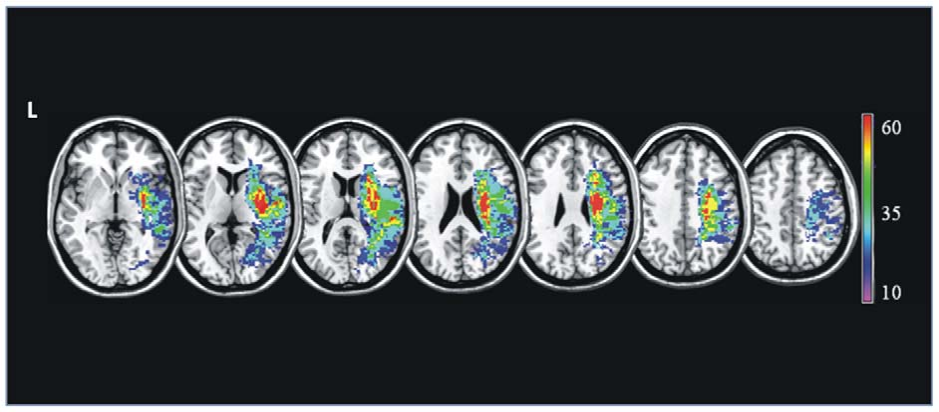
Lesion distribution. The damage evident in MRI images was reconstructed for each studied patient and plotted using MRIcro software. A T1-weighted template comprising 12 axial slices was used to demarcate lesions for every patient. The colour scale indicates the percentage of overlapping lesions across patients.

Aim of the current study was therefore to assess the presence of disconnection between hemispheres in patients with right brain damage, and its contribution in determining the presence and severity of neglect.

**Table 1:** Clinical assessment of visuospatial neglect.

	Mean (SD) score		Mean (SD) score
**BIT-C total score**	**11.1 (26.5)**	**BIT-B total score**	**56.4 (20.9)**
*BIT-C subtests*		*BIT-B subtests*	
Line crossing	32.3 (5.9)	Picture scanning	3.1 (2.6)
Letter cancellation	32.0 (4.3)	Telephone dialling	7.1 (2.7)
Star cancellation	43.7 (15.3)	Menu reading	6.4 (3.8)
Figure and shape copying	2.1 (1.3)	Article reading	5.9 (4.3)
Line bisection	5.7 (3.1)	Telling and setting the time	7.4 (1.7)
Representational drawing	2.3 (0.5)	Coin sorting	6.3 (3.1)
–		Address and sentence copying	7.6 (2.4)
–		Nap navigation	7.1 (3.1)
–		Card sorting	5.4 (2.5)

BIT-C  =  conventional scale of the Behavioural Inattention Test; BIT-B  =  behavioural scale of the Behavioural Inattention Test. See text for further details.

## Materials and Methods

### Study subjects

Eleven consecutive patients [F/M = 4/7; mean (SD) age: 59.7 (10.0) years] with clinical and radiological evidence of macroscopic damage to the right hemisphere, were recruited from the Specialist Rehabilitation Clinic of Santa Lucia Foundation (Rome, Italy). All patients had to be right-handed (as assessed by the Edinburg Handedness Inventory [25]) and to have suffered from an acute ischemic stroke over an interval of 1–6 months before enrolment. Exclusion criteria were: a previous history of cognitive decline, the absence of sensory deficits, and current impairment in cognitive domains other than visuospatial attention (see below). Major systemic, psychiatric and neurological illnesses other than stroke were carefully investigated in all patients by an expert stroke physician (G.K.) and excluded by standard clinical and laboratory assessments. Critical for this study, the presence and severity of left-side neglect was carefully quantified in each patient, as detailed below. Finally, all patients underwent MRI scanning at 3T, detailed below, and conventional MRI scans (i.e., dual echo and fluid attenuated inversion recovery [FLAIR]) were reviewed by an expert neuroradiologist. Patients were excluded in the presence of any macroscopic abnormality in addition to the right-hemispheric lesion. A group of 11 right-handed, age-and sex-matched healthy volunteers [F/M = 4/7; mean (SD) age: 59.3 (9.3) years] were also recruited for the study and served as controls. Healthy controls also underwent anamnestic interview and neurological examination to exclude major illnesses. On the basis of conventional MRI, subjects were excluded in the presence of any macroscopic abnormality. The current study was conformed to the ethical principles of the Helsinki Declaration, and received approval by the Ethics Committee of Santa Lucia Foundation. Written informed consent was obtained from all participants before study initiation.

**Figure 2:**
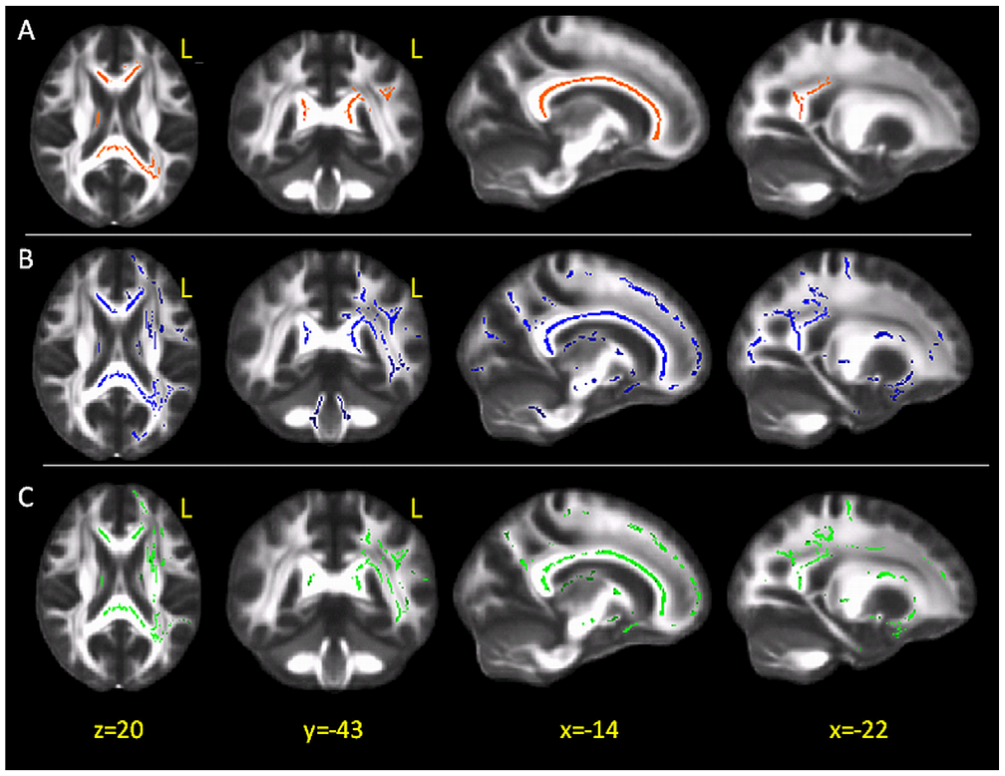
TBSS results in patients vs. controls: fractional anisotropy; radial diffusivity, and mean diffusivity. (A) Red voxels represent the areas where fractional anisotropy (FA) values of patients are significantly reduced with respect to those of healthy controls, overlaid onto the group-averaged FA image. FA values are significantly reduced in the corpus callosum and its projections on the parietal white matter. (B) Blue voxels indicate the areas of increased radial diffusivity in patients. The same sections as in panel A are shown to ease the comparion with FA results. (C) Green voxels indicate tracts where MD was increased in patients. Again, the same sections as in panels A and B are shown. L = left; x,y,z, indicate the MNI coordinates.

### Assessment of visuospatial neglect

The Behavioural Inattention Test (BIT) [26] was used to determine the presence and severity of hemispatial neglect. This is a comprehensive battery of tests for the evaluation of visuo-spatial deficits, which includes both conventional (BIT-C) and behavioural scales (BIT-B). The conventional tests include: 1) line crossing, 2) letter cancellation, 3) star cancellation, 4) figure and shape copying, 5) line bisection, and 6) representational drawing. The behavioural tests assess specific aspects of daily life activities, and include: 1) picture scanning, 2) telephone dialling, 3) menu reading, 4) article reading, 5) telling and setting the time, 6) coin sorting, 7) address and sentence copying, 8) map navigation and card sorting. The cut-off scores of normality for the conventional and behavioural tests are 129 (0–146, maximum score 146) and 67 (0–81, maximum score 81), respectively. Patients are classified as suffering from neglect when their score is below the cut-off score in either or both the BIT-C and BIT-B.

**Figure 3:**
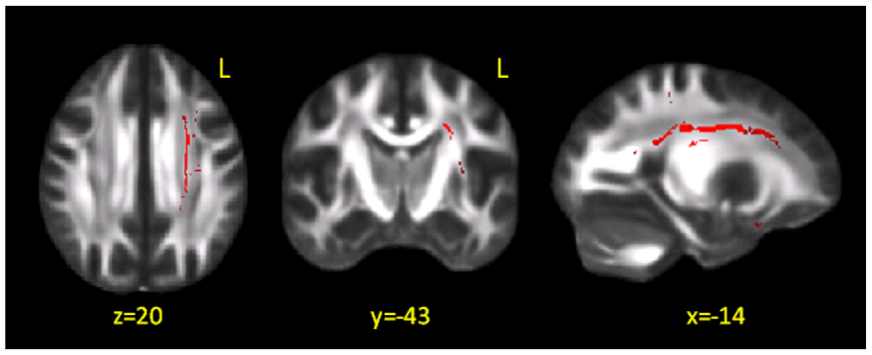
TBSS results in patients vs. controls: axial diffusivity. Red voxels indicate tracts where axial diffusivity (Dax) was found to be increased in patients compared to healthy subjects. These results are overlaid onto the group-averaged FA image. Changes were mainly located within the corona radiata, an area of crossing fibres, where the interpretation of changes in axial and radial diffusivity can be challenging. L = left; x, y, z, indicate the MNI coordinates.

### MRI acquisition

Brain imaging was obtained in a single session using a head-only 3.0T MR scanner (Siemens Magnetom Allegra, Siemens Medical Solutions, Erlangen, Germany). The acquisition protocol included the following sequences: 1) dual-echo turbo spin echo [TSE] (TR = 6190 ms, TE = 12/109 ms); 2) FLAIR (TR = 8170 ms, TE = 96 ms); 3) 3D Modified Driven Equilibrium Fourier Transform (MDEFT) scan (TR = 1338 ms, TE = 2,4 ms, Matrix = 256×224×176, in–plane FOV = 250×250 mm^2^, slice thickness = 1 mm); 4) Diffusion weighted twice-refocused SE EPI (TR = 7000 ms, TE = 85 ms, maximum b factor = 1000 smm^−2^, isotropic resolution 2.3 mm^3^). This sequence collects 7 images with no diffusion weighting (b_0_) and 61 images with diffusion gradients applied in 61 non-collinear directions.

**Figure 4:**
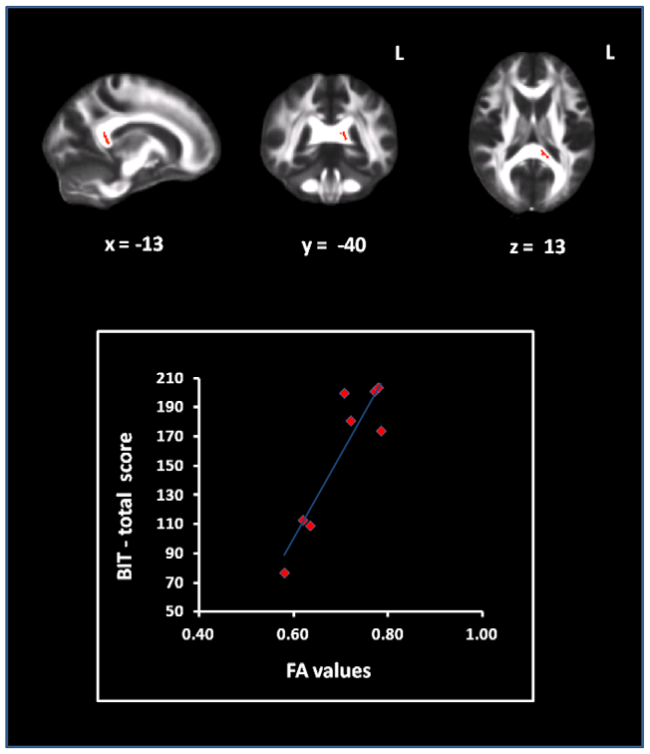
Correlation between patients' FA and BIT scores. In top panel it is illustrated the region of the posterior corpus callosum , whose fractional anisotropy (FA) value correlates with patients' performance at Behavioural Inattention test (BIT). In the bottom panel, the FA values extracted from that region are plotted, patient by patient, against their correspondent performance obtained at BIT. The post-hoc R value (Pearson correlation coefficient) was 0.91.

### MRI image analysis and statistics

#### Lesion assessment

For each patient, lesions were outlined on the MDEFT scans, using a semi-automated local thresholding contouring software (Jim 4.0, Xinapse System, Leicester, UK, http://www.xinapse.com/; accessed 2012 Sep 28). A binary lesion mask was obtained for every subject by setting all voxels within the lesion to 1 and the background to zero. The MDEFT scans were then normalised to standard space using tools from the FMRIB software library (FSL, www.fmrib.ox.ac.uk/fsl/; accessed 2012 Sep 28). First, the brain extraction tool (bet) was used to strip off the skull from every subject's MDEFT scan. Next, FLIRT [27] was used to compute the affine transformation that matches this skull-stripped image to the MNI brain atlas provided with FSL. Then, FNIRT [28] was used to compute the deformation field that warps the original MDEFT to the atlas, setting as starting estimate the affine transformation computed by FLIRT. Finally, the non-linear transformation was applied to the lesion binary mask. The masks from all subject were added and translated into a percentage unit to obtain a visual representation of the anatomical location of the lesions in the patient cohort (Figure 1). Lesion volumes were calculated from each patient's scan and correlated with the corresponding scores obtained at BIT, using the Spearman's Rank correlation test.

#### DTI and TBSS image processing

Diffusion data were processed using tools from FSL. After eddy currents correction the diffusion tensor was estimated in a voxel-wise fashion [29], and FA maps were derived for every subject. Maps of FA, MD, Dax and Drad were obtained.

FA maps were then fed into TBSS [22] to obtain a projection of all subjects' FA data onto a mean FA tract skeleton. Usually the skeleton is obtained by aligning every subject's FA image into a common space using non-linear registration, and then averaging the normalised images to create a mean FA map, which is finally thinned so that the FA skeleton represents the center of all tracts common to the group. Each subject's FA data is then projected onto the skeleton and voxel-wise statistics is carried out within the skeleton. The projection is achieved by searching perpendicular to the local skeleton structure for the maximum value in the subject's FA image. This maximum value is assumed to represent the nearest relevant tract centre. To avoid that the presence of lesion of the right hemisphere could affect the correct reconstruction of the skeleton, the TBSS pipeline was modified as follows. First, all FA images were affine registered to the FA template provided with FSL, masking out the lesion, which was outlined on b = 0 images. Once in standard space, the same portion of the right hemisphere (MNI coordinate x>18 mm) was removed from the images of all subjects. Note that in order to avoid edge effects along the midsagittal section of the corpus callosum, part of the right hemisphere (MNI coordinate x<18), unaffected by the lesions, was included in the analysis. The left hemisphere FA maps obtained through this procedure were transformed back into native space, and TBSS was performed as normal, but using a half-brain (including sagittal slices with x<18) template. The same transformation and projection were applied to MD, Dax and Drad maps. The healthy controls underwent an identical procedure in order to minimise any bias.

#### Statistical analysis

All TBSS voxel-wise statistics was carried out on the skeletonized images using the FSL tool “randomise”, which is based on permutation tests (500 iterations). A between-group comparison was first performed to identify regional FA, MD, Dax, and Drad differences, between patients and healthy controls. According to the specific processing, the analysis included all voxels in the left hemisphere and in the medial part of the corpus callosum. Then, in these same brain voxels, voxel-wise associations were investigated between patients' regional diffusion indices and total neuropsychological scores reported at BIT-C and BIT-B. For both, between-group comparison and correlation analyses, statistical significance was computed using permutation tests. A correction for multiple comparisons was obtained using the threshold-free cluster enhancement (TFCE) method [30]. P-values were accepted as significant if inferior to 0.05 after TFCE correction.

## Results

### Assessment of neglect

According to the criteria defined in the methods section, all patients were demonstrated to suffer from hemispatial neglect. As reported below, 3 patients were excluded from the analysis due to the poor quality of their MRI data (motion artifacts). From the remaining 8 patients, 1 reported scores above the cut-off normality in the BIT-B subtest only, 2 in the BIT-C subtest only, and 5 in both subtests. A detailed description of patients' performance at BIT is summarized in Table 1.

### MRI

Three out of 11 patients were excluded from image analysis for the poor quality of their MR images due to motion artefacts. According to the exclusion criteria, none of the patients who entered the analysis had any detectable macroscopic abnormality in the left hemisphere. None of the healthy controls' MRI scan revealed any macroscopic abnormality.

#### Lesion assessment

Figure 1 summarises the lesion data, which are presented here for completeness. Neglect patients typically had substantial lesions centred on right perisylvian structures, similar to many previous studies of neglect.

Correlation analysis between patients' lesion volumes and BIT scores did not return significant results (p = 0.6).

#### TBSS

The patient group compared to controls revealed a widespread reduction of regional FA in most of the left hemisphere tracts, with a predominant involvement of the corpus callosum and its projections towards the parietal WM (Figure 2A).

Widespread increases in Drad and in MD were also found in patients, located in the same tracts where FA was reduced and beyond (Figure 2B and 2C, respectively).

Areas of increased Dax were also found in patients, mainly located within the corona radiata (Figure 3).

No significant increases in FA, nor decreases in any of the other indices were observed.

Voxel-wise correlation analysis revealed a direct association between the patients' BIT scores and regional FA in a cluster located in the posterior portion of the corpus callosum, as shown in the top panel of Figure 4. Randomise provides the p-values but not the corresponding correlation coefficients. In order to have an estimate of the latter, we extracted, subject by subject, the mean FA of the significant cluster and computed post-hoc the Pearson correlation coefficient between this mean value and the BIT score. The bottom panel of Figure 4 shows the corresponding scatter plot. The correlation coefficient was 0.91. No associations were observed between the BIT scores and any of the other diffusion indices explored.

## Discussion

We recruited here a group of patients who suffered from an acute stroke of the RH and presented with symptoms of neglect. A detailed clinical assessment of neglect, based on the BIT, confirmed the presence of neglect in all recruited patients, with different degrees of severity. According to inclusion criteria, none of the patients had any macroscopic abnormality in the left hemisphere, as assessed on the T2 and FLAIR scans. Conversely, despite the absence of lesions, TBSS analysis was able to demonstrate subtle changes in the FA values along several WM tracts of the left hemisphere in patients. Wallerian degeneration is a well-described phenomenon, consisting of anterograde degeneration of axons and myelin sheaths after proximal axonal or cell body injury [31], [32]. In our patients, this reduction of FA fits with the expected evolution of the stroke lesion, which affected a proportion of neurons projecting from the right to the left hemisphere through the corpus callosum. Further analyses of Drad showed that this parameter was increased within and beyond the tracts where FA was found to be reduced. Conversely, Dax was found to be increased only in the corona radiata, an area where the crossing of several white matter pathways (corpus callosum, cortico-spinal tract, superior longitudinal fasciculus) is known to occur. Although changes in Drad and Dax have been associated with myelin and axonal damage, respectively [23 24], caution should be exercised when interpreting these indices in areas of crossing fibres [33]. Given these observations, we can therefore conclude that our data support the hypothesis that the main damage occurring in the left hemisphere of these patients is dominated by demyelination in the context of Wallerian degeneration phenomena. While the occurrence of Wallerian degeneration in one hemisphere can be expected in cases of macroscopic damage in the other hemisphere, an intriguing result of this study is that FA changes in the “healthy” hemisphere also accounted for the severity of neglect symptoms observed in our patients. Moreover, no association could be found between patients' severity of neglect symptoms and the volumetric assessment of the macroscopic lesions. When we performed the correlation analysis with the severity of neglect symptoms assessed by the BIT, TBSS analysis returned a well localized area of the posterior portion of the corpus callosum, which is known to transfer white matter fibers between the two homologues parietal cortices. The FA reduction is interpreted here as axonal demyelination/loss and, as a consequence, structural disconnection, correlated, without any a priori hypothesis on its anatomical location, with patients' performance at the BIT. This finding is strongly consistent with the hypothesis that neglect follows a disinhibition of parietal-frontal circuits of the left intact hemisphere (due to the release of right hemisphere control) in patients with neglect, as suggested by previous evidence based on TMS experiments [34]. We believe that the current finding provides novel anatomical evidence in support for a critical role of this inter-hemispheric network in neglect. On the other hand, against the “hemispheric specialization” theory of neglect [7], [8], no association could be found between patients' clinical severity and the volumetric assessment of their macroscopic lesions. Moreover, the data reported in the current study are not only interesting for clarifying the pathophysiology of neglect. There is a growing body of evidence that non-invasive brain stimulation techniques such as repetitive TMS or transcranial direct current stimulation (tDCS) may be used for therapeutic purposes [35]. For instance, relatively to neglect treatment, we recently reported that theta-burst stimulation, a form of rTMS, is able to accelerate recovery from neglect symptoms in stroke patients over a time window of few weeks [36]. In this context, but also in other clinical conditions of focal brain damage, the identification of the most critical networks producing specific symptoms may represent the target for neurophysiological treatments. This is particularly relevant in neurorehabilitation, for which non-invasive brain stimulation might contribute to improve the final outcome of the protocols currently in use.

Main limitation of the current study, which has to be considered as explorative, is the small sample size. Future studies on larger populations of patients are needed to confirm and extend our preliminary findings. On the other hand, the results presented here were obtained in a completely data-driven fashion, suggesting that the effect we observed in 8 patients only is likely to be rather strong.

In conclusion, this study provides new anatomical evidence supporting the notion that changes in right-left balance between the posterior parietal cortices rather than an isolated involvement of the right hemisphere can be critical for the occurrence of neglect symptoms, such as those explored by the BIT.
